# Draft Genome Sequences of Three Identified Rhizobacteria Associated with Maize Rhizosphere

**DOI:** 10.1128/mra.00970-21

**Published:** 2022-01-13

**Authors:** Iviwe Notununu, Deidre Alima Bregene van Wyk, Kazeem Adekunle Alayande, Ashira Roopnarain, Lucy Novungayo Moleleki, Rasheed Adegbola Adeleke

**Affiliations:** a Unit of Environmental Sciences and Management, North-West University, Potchefstroom, South Africa; b Microbiology and Environmental Biotechnology Research Group, Institute for Soil, Climate and Water—Agricultural Research Council, Pretoria, South Africa; c Department of Biochemistry, Genetics and Microbiology, Forestry and Agricultural Biotechnology Institute, University of Pretoria, Pretoria, South Africa; SIPBS, University of Strathclyde

## Abstract

In this study, we sequenced the entire genomes of three identified rhizobacterial strains associated with maize plantation. Genome annotation of the sequenced data revealed several putative growth-promoting proteins associated with the production of indoleacetic acids and siderophore, the assimilation of nitrogen, and phosphorus solubilization.

## ANNOUNCEMENT

Plant-microbe interactions are historically linked to improved plant growth and health. Several studies have, therefore, focused on rhizobacteria to determine their contribution toward improved nutrient acquisition, enhanced water retention, alleviation of abiotic stress, exclusion of plant pathogens, and ecofriendliness (1). Toward this end, the three rhizobacterial strains were recovered from maize-associated rhizosphere soil samples collected from Sheepmoor Village, Mpumalanga Province, South Africa. An air-dried soil sample (1 g) was serially diluted in sterile distilled water, spread-plated (100 μL) onto Pikovskaya’s agar medium containing tricalcium phosphate, and incubated at 30°C for 7 days. The three isolates were selected based on colonies that showed the formation of clear zones (halo zones), indicating phosphate solubilization potential (2).

A pure culture of each isolate was subcultured into tryptic soy broth (Sigma-Aldrich, Germany) and incubated at 35°C for 24 h. Then, the broth culture was centrifuged and washed in phosphate-buffered saline (PBS) at 5,000 rpm. Genomic DNA from the isolates was thereafter extracted using a DNA extraction kit (catalog number D6005; Zymo Research, USA) following the manufacturer’s instructions. The sequencing libraries were prepared using the Illumina DNA prep sample preparation kit (San Diego, CA), and the runs were performed on an Illumina MiSeq platform with v3 chemistry. The 2 × 300-bp paired-end read length was applied to generate 1,343,772 reads for Bacillus cereus 11MN1, 956,942 reads for Leclercia adecarboxylata 33MP1, and 693,584 reads for Leclercia adecarboxylata 36MP8.

Adapter regions and low-quality reads were filtered out of the sequence data using Trimmomatic v0.36 (3). The adapter sequences were clipped using a mismatch value of 2, a palindrome clip threshold of 30, and a simple clip threshold of 10. *De novo* genome assemblies were thereafter constructed using SPAdes v3.13.0 (4), while genome annotations were performed using the NCBI Prokaryotic Genome Annotation Pipeline (PGAP) v5.2 (5). The identities of the genomes were further confirmed through taxonomic assignments based on the Genome Taxonomy Database, using GTDB-Tk classify v6.1.0 (6), which revealed an average amino acid identity (AAI) of 99.3% for Bacillus cereus 11MN1 (reference genome, GCF_000007825.1) and average nucleotide identities (ANIs) of 98.66% and 98.64% for Leclercia adecarboxylata 33MP1 (reference genome, GCF_001515505.1) and Leclercia adecarboxylata 36MP8 (reference genome, GCF_001515505.1), respectively. The quality of the genome assemblies was equally assessed using CheckM v1.0.18 (7).

The basic features of each genome assembly are shown in Table 1. All three isolates possess protein encoding sequences, borne on multiple contigs, which are putatively responsible for the production of indoleacetic acids and siderophore, the assimilation of nitrogen, and the solubilization of phosphorus.

**TABLE 1:** Basic characteristics of the genome assemblies of the three rhizobacterial strains

Bacterial strain	Assembly size (bp)	No. of contigs	Completeness (%)	G+C content (%)	*N*_50_ (bp)	*L*_50_	No. of CDS (total)^*a*^	No. of CDS (proteins)	No. of pseudogenes	No. of RNAs (total)
Bacillus cereus 11MN1	6,217,338	45	99.43	34.72	584,692	4	6,134	5,958	176	119
Leclercia adecarboxylata 33MP1	5,122,041	93	99.9	55.59	268,390	6	4,816	4,724	92	114
Leclercia adecarboxylata 36MP8	5,061,295	79	99.9	55.63	228,037	7	4,749	4,667	82	114

a CDS, coding DNA sequences.

The completeness of the genomes was determined using CheckM v1.0.18.

### Data availability.

All data were deposited under the GenBank BioProject accession number PRJNA761544. The whole-genome shotgun projects for Bacillus cereus 11NM1, Leclercia adecarboxylata 33MP1, and Leclercia adecarboxylata 36MP8 were deposited at DDBJ/ENA/GenBank under the accession numbers JAIPUK000000000.1, JAIPRI000000000.1, and JAIPRJ000000000.1, respectively. The SRA accession numbers for the raw reads are SRX12096037, SRX12099443, and SRX12100642, respectively.
